# Exploring Safety–Stability Tradeoffs in Cooperative CAV Platoon Controls with Bidirectional Impacts

**DOI:** 10.3390/s24051614

**Published:** 2024-03-01

**Authors:** Yu Wei, Xiaozheng He

**Affiliations:** Department of Civil and Environmental Engineering, Rensselaer Polytechnic Institute, Troy, NY 12180, USA; yu.ryan.wei@gmail.com

**Keywords:** CAV platoon, bidirectional sensing and communication, phase shift, safety, stability

## Abstract

Advanced sensing technologies and communication capabilities of Connected and Autonomous Vehicles (CAVs) empower them to capture the dynamics of surrounding vehicles, including speeds and positions of those behind, enabling judicious responsive maneuvers. The acquired dynamics information of vehicles spurred the development of various cooperative platoon controls, particularly designed to enhance platoon stability with reduced spacing for reliable roadway capacity increase. These controls leverage abundant information transmitted through various communication topologies. Despite these advancements, the impact of different vehicle dynamics information on platoon safety remains underexplored, as current research predominantly focuses on stability analysis. This knowledge gap highlights the critical need for further investigation into how diverse vehicle dynamics information influences platoon safety. To address this gap, this research introduces a novel framework based on the concept of phase shift, aiming to scrutinize the tradeoffs between the safety and stability of CAV platoons formed upon bidirectional information flow topology. Our investigation focuses on platoon controls built upon bidirectional information flow topologies using diverse dynamics information of vehicles. Our research findings emphasize that the integration of various types of information into CAV platoon controls does not universally yield benefits. Specifically, incorporating spacing information can enhance both platoon safety and string stability. In contrast, velocity difference information can improve either safety or string stability, but not both simultaneously. These findings offer valuable insights into the formulation of CAV platoon control principles built upon diverse communication topologies. This research contributes a nuanced understanding of the intricate interplay between safety and stability in CAV platoons, emphasizing the importance of information dynamics in shaping effective control strategies.

## 1. Introduction

Platoon control aims to minimize speed variations among vehicles while ensuring consistent and secure spacing between them [1]. This approach offers a promising solution to several pressing concerns of today’s road transportation due to its potential to increase highway capacity, enhance safety, and reduce fuel consumption [2]. The recent advent of CAV technologies has received much attention in platoon control, largely owing to the pivotal role of communication and information technologies, including advanced sensors, 5G network and a variety of communication protocols [3,4]. These technologies significantly enhance platoon safety and stability [5,6].

Leveraging communication technology, platoon controls can be developed based on Cooperative Adaptive Cruise Control (CACC), typically comprising four components [1,2]: (1) vehicle dynamics, especially longitudinal vehicle dynamics, which depict the behavior of each vehicle in the longitudinal direction, (2) information exchange that describes how vehicles communicate with other vehicles, including the exchanged information and information flow topology (IFT) that determines the configuration of V2V communication links in vehicle platoon, (3) a controller that uses information from other vehicles in the platoon to devise control strategies, and (4) formation geometry that describes the spatial arrangement of vehicles within the platoon.

Among the four components, information exchange is crucial, facilitated by advanced information and communication technologies that support different information flow topologies for effective communication in CAV platoon control [7]. Various information topologies offer both benefits and challenges to the design and analysis of multi-vehicle systems, such as predecessor following topology, predecessor–leader following topology, multiple predecessor following topology, and bidirectional topology [8]. Among them, bidirectional topology stands out as popular and extensively applied in various studies due to its simple structure. In bidirectional topology, the subject vehicle adjusts its velocity by not only following the preceding vehicle but also taking into account the dynamics of the following vehicle.

CAV platoon controls employing bidirectional topology are also referred to as bidirectional car-following control models. Existing bidirectional models primarily aim to improve overall platoon stability by integrating abundant information from vehicles traveling behind. However, the incorporation of increasingly complex layers of information into these models comes with its drawbacks. One significant oversight in this pursuit of enhanced stability is the neglect of safety analysis—an essential aspect that remains underexplored. The emphasis on stability often overshadows the potential safety implications of adding complexity to control protocols. It is crucial to acknowledge that the reception of back-looking information from following vehicles can affect the dynamics of preceding vehicles, leading to significant safety concerns. Current studies are examining the tradeoff between safety and stability in automated vehicles [9], but the impact of bidirectional communication on this tradeoff is not thoroughly understood yet. Additionally, there is a lack of comprehensive investigation into the diverse effects resulting from different types of information in current research.

To address these research gaps, this study introduces a novel framework utilizing the concept of phase shift to examine the influence of back-looking information on platoons considering both stability and safety aspects. Employing the proposed framework, this research analyzes how spacing information and velocity difference information affect CAV platoon safety and stability. Theoretical analysis reveals that incorporating spacing information of the following vehicle improves both platoon string stability and safety. In contrast, adopting velocity difference information enhances either safety or stability, but not both simultaneously. These theoretical findings are validated through numerical experiments conducted on both linear and non-linear car-following models.

The remainder of this research is organized as follows. The subsequent section is the literature review of existing bidirectional models, offering a comprehensive background. Section 3 presents the methodology which encompasses an illustration of platoon vehicle dynamics with bidirectional information flow topology. Then, the proposed phase-shift-based framework is introduced, followed by an analysis of the safety conditions and the derivation of string stability for two types of information. Additionally, the tradeoff between safety and stability is examined. Section 4 and Section 5 validate the theoretical and numerical analysis using specific linear and non-linear car-following models. The final section summarizes the main findings and offers recommendations for future research.

## 2. Literature Review

The exchange of vehicle dynamics information is pivotal for CAV platoon control. The most commonly used vehicle dynamics information includes velocity and spacing [10,11,12]. Other types of vehicle information are also considered, such as acceleration [13], traffic jerk [14,15], visual angle [16], and electronic throttle opening angle [17,18]. Abundant vehicle information can facilitate platoon control design to achieve better platoon performances in a coordinated manner. 

This research focuses on bidirectional car-following control models. Existing bidirectional models primarily depend on two prevalent types of vehicle dynamic information to maintain harmonized speeds with constant time headways: spacing, which is the distance between the target vehicle and the following vehicle, and the velocity difference between the target and following vehicles [19]. Table 1 categorizes existing bidirectional car-following control models into three categories by types of back-looking information utilized, including spacing information only, velocity difference information only and both types of information.

The first category of bidirectional car-following control models utilizes spacing information only. Spacing information is widely used since it can be easily measured by sensors. For example, Nakayama et al. extended the optimal velocity model (OVM) by introducing a back-looking optimal velocity function. The modified model takes into account one preceding and one following vehicle, and it has been shown to improve traffic stability compared to the traditional OVM model [20]. Hasebe et al. extended the OVM model by considering the headway of multiple preceding and following vehicles. The study examined the linear stability of the modified model, revealing that it displayed dynamic properties capable of mitigating velocity fluctuations [21]. Ge et al. proposed an extension of the OVM model that takes into account an arbitrary number of vehicles ahead and one vehicle following. Linear stability analysis was conducted to demonstrate the enhanced stabilizing effect [22]. Chen et al. extended the full velocity difference (FVD) model by considering the driver’s sensory memory and the back-looking effect [23]. And Hou et al. further incorporated the bidirectional FVD models with the driver’s visual angle [24]. Ma et al. improved the FVD model by accounting for the time-delayed velocity difference and back-looking effect [25]. Yi et al. introduced a new bidirectional distance-balanced model that was built upon the Intelligent Driver Model (IDM). This model aims to balance the distance between the host vehicle and its nearest preceding and following vehicles. The authors conducted analyses on the local stability and string stability of the proposed model [26].

The second category solely utilizes velocity difference information. Models in this category are relatively rare. Herman et al. were the first to propose a bidirectional car-following control model using velocity differences, taking into account both the velocity difference between the target vehicle and the preceding vehicle and the velocity difference between the target vehicle and the following vehicle. It was found that the local and string stability conditions improved as the weights of the forward-looking and back-looking decisions increased [27].

Research in the third category combines spacing and velocity difference information when designing car-following control models. Yang et al. presented a new extension of the OVM model that considers an arbitrary number of preceding and following vehicles. The study found that the back-looking effect can help to stabilize traffic flow [28]. Hu et al. proposed an extension of the OVM model that considers bidirectional visual fields and multiple anticipations. A stability analysis of the model revealed that multiple anticipations can enhance the stability of traffic flow. The results demonstrated that the extended model is capable of reproducing the local clustering phenomenon observed in the traffic flow [19]. Sun et al. proposed a bidirectional car-following model based on the FVD model, which takes into account multiple preceding vehicles and only one following vehicle [29]. Yang et al. introduced a bidirectional Gipps’ model and investigated the linear stability of traffic flow. The results indicated that the back-looking behavior has three types of effects on traffic flow stability: stabilizing, destabilizing, and generating non-physical phenomena, which are more complex than the effects derived from OVM-based bidirectional models. Furthermore, the study discovered that drivers with shorter reaction times and larger additional delays can contribute to stabilizing traffic flow [30]. Horn and Wang incorporated the back-looking effect into Helly’s car-following model. This research developed the damped wave equation for stability analysis under bidirectional control, where the ‘damping’ component is critical to the dissipation of perturbations [31]. Yi et al. proposed an extended bidirectional car-following model based on the IDM in the CAV environment, which considers the desired distance of the following vehicle as a control term. The study investigated the linear stability of the model, and theoretical and simulation results indicated that bidirectional IDM improves string stability. Furthermore, stability can be further enhanced by increasing the proportion of the desired distance of the following vehicle [32].

## 3. Methodology

This section is structured to examine key components vital to our research. Section 3.1 lays the groundwork by introducing vehicle longitudinal dynamics models with bidirectional information flow. Subsequent Section 3.2 and Section 3.3 introduce phase shift effects and employ a framework rooted in this concept to analyze the interconnected behavior of vehicles within a platoon under bidirectional communication. Building upon this groundwork, the study further evaluates the rear-end collision risk and conducts a stability analysis in Section 3.4 and Section 3.5, respectively. And culminating findings are presented in Section 3.6.

### 3.1. Vehicle Longitudinal Dynamics Models with Bidirectional Information Flow

In CAV environment, the dissemination of real-time data is essential for ensuring the safety and stability of vehicles within a platoon. This critical process is supported by advanced sensor and communication technologies, which are essential for enabling automated vehicles to share information seamlessly. Key sensor devices include RADAR, cameras, and LIDAR, etc. Furthermore, advanced communication technologies, including Dedicated Short-Range Communications (DSRC) and 5G, are indispensable for facilitating Vehicle-to-Vehicle (V2V), Vehicle-to-Infrastructure (V2I), and Vehicle-to-Everything (V2X) communications [33].

Meanwhile, the effectiveness of information delivery within this communication network heavily relies on communication protocols. These protocols are designed to ensure that necessary information is disseminated to the intended vehicle in a reliable and low-latency manner [34]. The development and implementation of a variety of communication protocols have been proposed to significantly improve the safety and stability of vehicular platoons [3,4,35].

The integration of information technologies and protocols enables CAVs to exchange information seamlessly with each other. In a CAV platoon, the control system operates the vehicle using locally sensed information and information shared among vehicles [36]. A car-following model can be used to describe the vehicle’s longitudinal dynamics. Note that, at present, our analysis is confined to a one-dimensional perspective. The car-following model, along with our safety and stability analysis, does not account for lateral dynamics, such as left and right turns. In the literature, continuous-time car-following models have a generalized form, which can be expressed below [37,38]; we adapted this format to include additional information:

(1) $${\dot{x}}_{n}(t)=v_{n}(t)\;{\dot{v}}_{n}(t)=f(s_{n}\left(t\right),\;v_{n}\left(t\right),\;\Delta v_{n}\left(t\right),\;K(t))$$

where ${\dot{v}}_{n}(t)$ is the control variable, which represents the acceleration (or deceleration) of the *n*th vehicle at time *t*. This variable is what the control system aims to adjust through the function. $x_{n}$ and $v_{n}$ represent the position and velocity of vehicle n, $s_{n}=x_{n-1}-x_{n}-l_{n-1}$ represents the net distance between vehicle n and vehicle n−1, $l_{n-1}$ indicates the length of vehicle n−1, and $\Delta v_{n}=v_{n}-v_{n-1}$ represents the velocity difference between vehicle n and vehicle n−1. In this study, we apply a broader interpretation of net distance, referring to it as “spacing.” In Equation (1), K(t) symbolizes additional information, encompassing vehicle dynamic data from the following vehicle in bidirectional car-following control, like velocity or spacing. Moreover, it extends to represent various other data types, such as acceleration [13], traffic jerk [14,15], visual angle [16], and electronic throttle opening angle [17,18].

**Figure 1:**
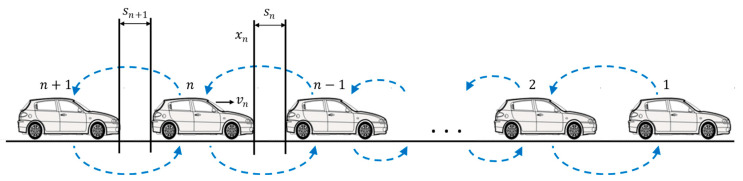
Illustration of the platooned vehicles using bidirectional communication.

For platoon control with bidirectional information flow topology, the target vehicle reacts not only to the preceding vehicle but also to the dynamics of the following vehicle to adjust its speed, as shown in Figure 1. Typically, bidirectional control utilizes two types of back-looking information from the following vehicle, i.e., spacing $s_{n+1}$ and velocity difference $\Delta v_{n+1}$ information. We denote as $s_{n+1}=x_{n}-x_{n+1}-l_{n}$ the spacing between vehicle n+1 and vehicle n, and as $\Delta v_{n+1}=v_{n+1}-v_{n}$ the velocity difference between vehicle n+1 and vehicle n. The car-following models with bidirectional communication topology have a generic form as in Equation (2). If only spacing information $s_{n+1}$ is utilized, the resulting model is formulated in Equation (3) and referred to in this research as the spacing bidirectional control model.

(2) $${\dot{x}}_{n}(t)=v_{n}(t)\;{\dot{v}}_{n}\left(t\right)=f\left(s_{n}\left(t\right),\;v_{n}\left(t\right),\;\Delta v_{n}\left(t\right),\;s_{n+1}\left(t\right),\Delta v_{n+1}\left(t\right)\right)$$

If only spacing information $s_{n+1}$ is utilized, the resulting model is formulated in Equation (3) and referred to in this research as the spacing bidirectional control model.

(3) $${\dot{v}}_{n}(t)=f(s_{n}\left(t\right),\;v_{n}\left(t\right),\;\Delta v_{n}\left(t\right),\;s_{n+1}\left(t\right))$$

If only velocity difference information $\Delta v_{n+1}$ is utilized, the resulting model is formulated in Equation (4) and referred to in this research as the velocity difference bidirectional control model.

(4) $${\dot{v}}_{n}(t)=f(s_{n}(t),\;v_{n}(t),\;\Delta v_{n}(t),\;\Delta v_{n+1}(t))$$

### 3.2. Phase Shift Effects

In a CAV system, the dynamics of vehicles are impacted by several factors, including the vehicle’s control mechanisms, types of exchanged information, communication delays, packet losses, and the design of information flow topology. For instance, in CACC systems that utilize bidirectional information flow topology, vehicles take into account information from the following vehicle, which affects the dynamics of the preceding vehicles, potentially giving rise to substantial safety concerns. However, there has not yet been a generalized model to comprehensively assess such impacts on vehicle dynamics.

Inspired by our recent discovery that a perturbed vehicular platoon exhibits periodic oscillatory dynamics characterized by inherent frequency [39,40,41], this research proposed a generalized analysis model based on the concept of phase shift to capture the interactions among connected vehicles’ dynamics. Our research demonstrated that the oscillation dynamics of a perturbed vehicle platoon can be described by a second-order non-homogeneous ordinary differential equation (ODE), resulting in periodic oscillations propagating within the platoon. In the context of a CAV platoon employing bidirectional communication, the effects of perturbations can be transmitted both forward and backward simultaneously, leading to an overlapping effect of two periodic oscillations. This overlapping effect can be analyzed using the concept of phase shift. 

**Figure 2:**
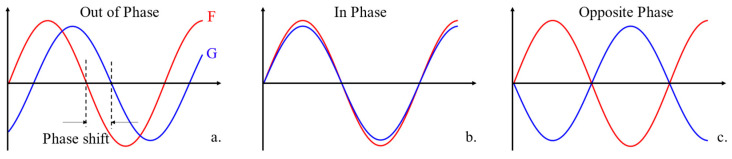
Demonstration of phase shift. (**a**) Phase shift. (**b**) In Phase. (**c**) Opposite Phase.

The concept of phase shift is commonly used in physics, for example, in acoustics and optics [42,43], to describe the differences, $\varphi \left(t\right)=\phi_{G}\left(t\right)-\phi_{F}\left(t\right),$ between the phases of two periodic signals *F* and *G*, as depicted in Figure 2a. Specifically, when the difference is zero, the two signals are in phase (IP), as shown in Figure 2b, indicating perfect synchronization. On the contrary, when the difference is not zero, implying $\varphi \left(t\right)\neq 0$, the two signals are termed out-of-phase, as illustrated in Figure 2a. For sinusoidal signals, when difference $\varphi \left(t\right)$ is 180°, the two phases are opposite, defined as opposite phase (OP), as illustrated in Figure 2c. 

The concept of phase shift provides a valuable framework for understanding the interactions among vehicles through information exchange. In this framework, we distinguish between different vehicles involved in the information exchange process by categorizing them as either the “source vehicle” or the “target vehicle”. The “source vehicle,” represented as signal *F* in Figure 2, refers to the vehicle that transmits stimulus information. On the other hand, the “target vehicle,” denoted as signal *G* in Figure 2, refers to the vehicle that receives this stimulus information and responds accordingly.

Phase shift plays a crucial role in depicting target vehicle response to stimulus information, encompassing factors such as latency and response patterns. To represent latency, let us consider ${\dot{v}}_{n}(t)$, which represents vehicle dynamics with delay $t_{d}$ indicating the latency in the target vehicle’s response to perturbation from the source vehicle. In this context, phase shift $\varphi \left(t\right)$ can serve as a representation of latency $t_{d}$. The relationship between phase shift $\varphi \left(t\right)$ and latency $t_{d}$ is associated with the wavelength of the resulting platoon oscillation, which can be analytically derived using the corresponding ODE [41]. Furthermore, phase shift can also be utilized in depicting response patterns. When phase shift $\varphi \left(t\right)=0$ and there is no latency ($t_{d}=0$), the dynamics of the two adjacent vehicles synchronize their responses to perturbation. If the target vehicle responds to the source vehicle in a synchronized manner, this can be denoted as “in-phase.” On the contrary, if the response is unsynchronized with phase shift $\varphi \left(t\right)\neq 0$, it is denoted as being “out of phase”. If the response displays an adverse manner, it is characterized as “opposite-phase.”

### 3.3. Phase Shift Effects in Bidirectional Communication

**Figure 3:**
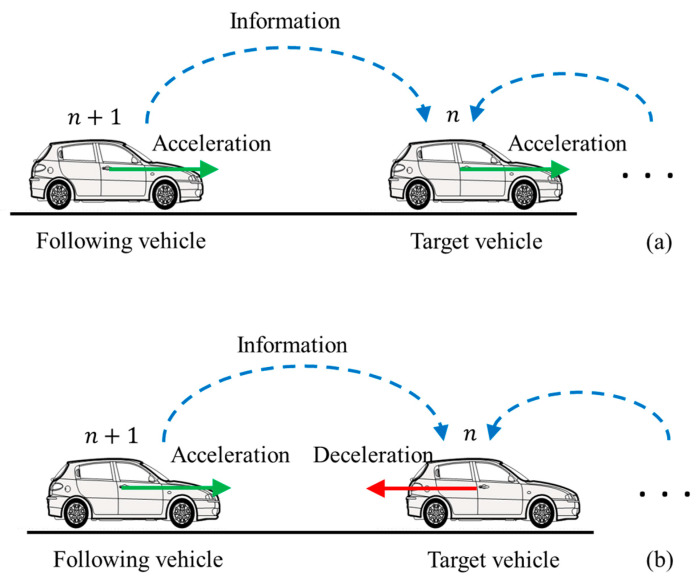
Illustration of the (**a**) in-phase effect (**b**) opposite-phase effect.

The present research employs the proposed framework centered around phase shift to characterize the interconnected behavior of the following vehicle and the target vehicle in a platoon under bidirectional communication topology. In this scenario, the following vehicle serves as the source vehicle, transmitting stimulus information, while the target vehicle receives this stimulus information and responds accordingly. As an initial step to analyze safety impacts, this research narrows the focus to two specific and extreme cases of phase shift without considering communication latency: the in-phase (IP) effect and the opposite-phase (OP) effect. The in-phase (IP) effect indicates a synchronized manner between the following vehicle and the target vehicle. For instance, if the following vehicle accelerates abruptly, the target vehicle also accelerates, as depicted in Figure 3a. In contrast, the opposite-phase (OP) effect reflects an adverse manner. For instance, if the following vehicle accelerates abruptly, the target vehicle decelerates to adjust its speed, as shown in Figure 3b.

The rest of this section outlines the conditions for the IP and OP effects in the spacing bidirectional control model and the velocity difference bidirectional control model. Considering that a platoon of CAVs runs on a single lane with all vehicles in an equilibrium state, i.e., maintaining the same spacing and velocity, perturbation is introduced to the following vehicle n+1 at time t, resulting in a deviation denoted as velocity deviation $\mu_{n+1}\left(t\right)=v_{n+1}-v_{n}$ and spacing deviation $\varphi_{n+1}\left(t\right)=s_{n+1}(t)-s_{e}$, where $s_{e}$ represents spacing at the equilibrium state. We have

(5) $$\mu_{n+1}\left(t\right)=-{\dot{\varphi }}_{n+1}\left(t\right)\;\varphi_{n+1}\left(t\right)*\mu_{n+1}\left(t\right)<0$$

In a bidirectional control model, the velocity deviation of the following vehicle n+1 prompts the target vehicle n to adjust its speed. By taking the first-order Taylor expansion of Equation (3) of the spacing bidirectional control model, we can express the acceleration of target vehicle n as shown in Equation (6).

(6) $${\dot{v}}_{n}\left(t\right)=f_{s_{n+1}}\varphi_{n+1}\left(t\right)$$

By taking the first-order Taylor expansion of Equation (4) of the velocity difference bidirectional control model, the acceleration of the target vehicle n can be expressed as in Equation (7).

(7) $${\dot{v}}_{n}\left(t\right)=f_{{\Delta v}_{n+1}}\mu_{n+1}(t)$$

where $f_{s_{n+1}}={\left.\frac{\partial f_{n}}{\partial s_{n+1}}\right|}_{\left(\bar{v},\;\bar{s}\right)}$ represents velocity differential with respect to spacing change, and $f_{{\Delta v}_{n+1}}={\left.\frac{\partial f_{n}}{\partial {\Delta v}_{n+1}}\right|}_{\left(\bar{v},\;\bar{s}\right)}$ represents velocity differential with respect to velocity difference change.

Let us first concentrate on the IP effect. When $\varphi_{n+1}\left(t\right)\;*\;{\dot{v}}_{n}\left(t\right)<0$ or $\mu_{n+1}\left(t\right)\;*\;{\dot{v}}_{n}\left(t\right)>0$, it implies that if vehicle n + 1 accelerates or decelerates, target vehicle n will also accelerate or decelerate, as demonstrated in Figure 3a. The IP effect is determined by the positivity or negativity of $f_{s_{n+1}}$ and $f_{{\Delta v}_{n+1}}$. If $f_{s_{n+1}}<0$, then $\varphi_{n+1}\left(t\right)\;*\;{\dot{v}}_{n}\left(t\right)<0$. If $f_{{\Delta v}_{n+1}}>0$, then $\mu_{n+1}\left(t\right)\;*\;{\dot{v}}_{n}\left(t\right)>0$.

Correspondingly, when $\varphi_{n+1}\left(t\right)\;*\;{\dot{v}}_{n}\left(t\right)>0$ or $\mu_{n+1}\left(t\right)\;*\;{\dot{v}}_{n}\left(t\right)<0$, it means that if vehicle n+1 accelerates or decelerates, the target vehicle n will respond oppositely by decelerating or accelerating; this phenomenon is denoted as the OP effect, as illustrated in Figure 3b. The OP effect is determined by the positivity or negativity of $f_{s_{n+1}}$ and $f_{{\Delta v}_{n+1}}$. If $f_{s_{n+1}}>0$, then $\varphi_{n+1}\left(t\right)\;*\;{\dot{v}}_{n}\left(t\right)>0$. If $f_{{\Delta v}_{n+1}}<0$, then $\mu_{n+1}\left(t\right)\;*\;{\dot{v}}_{n}\left(t\right)<0$. The OP effect increases the risk of rear-end collisions since the target vehicle and the following vehicle exhibit opposite behaviors, especially when the following vehicle experiences sudden acceleration.

Factors $f_{s_{n+1}}$, $f_{{\Delta v}_{n+1}}$ are identified as crucial elements that dictate whether a platoon operates in in-phase (IP) or opposite-phase (OP) modes, a distinction that is pivotal for understanding the platoon’s reaction to internal disturbances. This identification of key factors is fundamental to our further analysis concerning safety and string stability in platoon dynamics. We delve deeper into these aspects in Equations (10) and (12), which relate to safety, and Equations (25) and (27), which concern string stability.

### 3.4. Assessment of Rear-End Collision Risk

Rear-end collisions are prevalent on freeways, representing one of the most common types of accidents [44]. The advent of vehicle-to-vehicle (V2V) and vehicle-to-infrastructure (V2I) communications has made it possible to use individual vehicle information to assess collision risks and enhance traffic safety. Therefore, this section focuses on assessing the collision risk associated with the bidirectional control model.

In this research, surrogate safety measures (SSMs) are utilized to evaluate collision risks. SSMs are safety performance indicators that estimate accident risks based on microscopic traffic parameters like speed, space headway, and time headway [44]. Several SSMs have been developed for estimating collision risks. Time to collision (TTC) is one of the most used SSMs. The concept of TTC, introduced in [45], refers to the time remaining until collision occurs between the leading and the following vehicle if velocity difference is maintained. 

This research adopts TTC as a metric to analyze the risk of rear-end collisions. Given that perturbation is introduced to the following vehicle n+1 at time t, it impacts the dynamics of vehicle n due to the bidirectional control mechanism. Our analysis specifically focuses on the collision risk between vehicle n+1 and vehicle n, as this pair presents a heightened risk of collision, particularly when the following vehicle n+1 undergoes sudden acceleration. The TTC for vehicle n+1 is calculated as follows:

(8) $$TTC_{n+1}\left(t\right)=\left\{\begin{array}{c} \frac{x_{n}\left(t\right)-x_{n+1}\left(t\right)-l_{n}}{v_{n+1}\left(t\right)-v_{n}(t)},\;if\;v_{n+1}\left(t\right)>v_{n}(t)\\ \infty ,\;if\;v_{n+1}\left(t\right)<v_{n}(t) \end{array}\right.$$

where $TTC_{n+1}\left(t\right)$ denotes the TTC value of vehicle n+1 at time t, $x_{n}$ and $x_{n+1}$ are the positions of vehicles n and n+1, $v_{n}$ and $v_{n+1}$ are the velocities of vehicles n and n+1, and $l_{n}$ is the length of vehicle n. A smaller TTC value indicates a higher risk of collision.

As discussed earlier, the OP effect increases the risk of collisions, especially when the following vehicle experiences sudden acceleration. Thus, we consider the perturbation in Section 3.2 when $\mu_{n+1}\left(t\right)>0$ ($\varphi_{n+1}\left(t\right)<0$). The TTC of vehicle n+1 after time Δt for spacing and velocity difference bidirectional control can be represented using Equations (9) and (11), respectively.

For the spacing bidirectional control model, $TTC_{n+1}\left(t+\Delta t\right)$, denoted as $TTC_{n+1}^{s}\left(t+\Delta t\right)$, is expressed as follows:

(9) $$TTC_{n+1}^{s}\left(t+\Delta t\right)=\frac{x_{n}\left(t+\Delta t\right)-x_{n+1}\left(t+\Delta t\right)-l_{n}}{v_{n+1}\left(t+\Delta t\right)-v_{n}\left(t+\Delta t\right)}\;=\frac{\varphi_{n+1}\left(t+\Delta t\right)+s_{e}}{\mu_{n+1}\left(t+\Delta t\right)-f_{s_{n+1}}\varphi_{n+1}\left(t\right)\Delta t}$$

By taking partial derivation of Equation (9) with respect to $f_{s_{n+1}}$, we obtain the following equation:

(10) $${\left.\frac{\partial TTC_{n+1}^{s}\left(t+\Delta t\right)}{\partial f_{s_{n+1}}}\right|}_{\left(\bar{v},\;\bar{s}\right)}=\frac{(\varphi_{n+1}\left(t+\Delta t\right)+s_{e})\varphi_{n+1}\left(t\right)\Delta t}{{{(\mu }_{n+1}\left(t+\Delta t\right)-f_{s_{n+1}}\varphi_{n+1}\left(t\right)\Delta t)}^{2}}$$

Given that perturbation $\varphi_{n+1}\left(t\right)$ is small and does not result in an immediate collision, we can infer that $\varphi_{n+1}\left(t+\Delta t\right)+s_{e}>0$. Thus, we can conclude that $\frac{\partial TTC_{n+1}\left(t+\Delta t\right)}{\partial f_{s_{n+1}}}<0$.

Since $\frac{\partial TTC_{n+1}^{s}\left(t+\Delta t\right)}{\partial f_{s_{n+1}}}<0$, it can be observed that when $f_{s_{n+1}}<0$ (IP), $TTC_{n+1}\left(t+\Delta t\right)$ increases, resulting in lower collision risk and improved safety. On the contrary, when $f_{s_{n+1}}>0$ (OP), $TTC_{n+1}\left(t+\Delta t\right)$ decreases, leading to higher collision risk.

For the velocity difference bidirectional control model, $TTC_{n+1}\left(t+\Delta t\right)$, denoted as $TTC_{n+1}^{\Delta v}\left(t+\Delta t\right)$, is expressed as follows:

(11) $$TTC_{n+1}^{\Delta v}\left(t+\Delta t\right)=\frac{\varphi_{n+1}\left(t+\Delta t\right)+s_{e}}{\mu_{n+1}\left(t+\Delta t\right)-f_{\Delta v_{n+1}}\mu_{n+1}(t)\Delta t}$$

By taking partial derivation of Equation (11) with respect to $f_{n+1}^{\Delta v}$, we obtain the following equation:

(12) $${\left.\frac{\partial TTC_{n+1}^{\Delta v}\left(t+\Delta t\right)}{\partial f_{\Delta v_{n+1}}}\right|}_{\left(\bar{v},\;\bar{s}\right)}=\frac{(\varphi_{n+1}\left(t+\Delta t\right)+s_{e})\mu_{n+1}(t)\Delta t}{{{(\mu }_{n+1}\left(t+\Delta t\right)-f_{\Delta v_{n+1}}\mu_{n+1}(t)\Delta t)}^{2}}$$

Since $\frac{\partial TTC_{n+1}^{\Delta v}\left(t+\Delta t\right)}{\partial f_{\Delta v_{n+1}}}>0$, it can be observed that when $f_{\Delta v_{n+1}}>0$ (IP), $TTC_{n+1}^{\Delta v}\left(t+\Delta t\right)$ increases, resulting in lower collision risk and improved safety. On the contrary, when $f_{\Delta v_{n+1}}<0$ (OP), $TTC_{n+1}^{\Delta v}\left(t+\Delta t\right)$ decreases, leading to higher collision risk.

Based on the analysis presented above, we can conclude that for both the spacing and velocity difference bidirectional control models, the IP effect decreases rear-end collision risk, while the OP effect increases the risk.

### 3.5. String Stability Analysis

This section derives the string stability of the bidirectional control model. String stability represents the ability of one vehicle to withstand small perturbations and progress to the steady state where vehicles travel with an identical gap and speed in homogenous traffic [46]. This research adopts the linear stability analysis method described in [21,32,47,48] to analyze string stability. We first derive the stability condition for the generic model, which adopts both spacing and velocity difference information in Equation (2). We then derive the stability condition for the specific models that only use one type of information.

Considering a platoon with *N* vehicles, in the steady state, each vehicle can be represented as

(13) $${\bar{x}}_{n}\left(t\right)=\left(N-n\right)h_{0}+\bar{v}t,\;\;\;\;\;\;\;\;\;\;\;\;\;n=1,2\ldots ,N$$

where $h_{0}$ denotes the average headway of adjacent vehicles in the steady state in homogenous traffic, $\bar{v}$ represents the velocity of vehicles in the steady state, and ${\bar{x}}_{n}\left(t\right)$ is the location of vehicle n at time t.

We assume that a small perturbation affects the steady state solution of vehicle n at time t. We denote the perturbation by $y_{n}\left(t\right)$ that has a linear Fourier-mode expansion,

(14) $$y_{n}\left(t\right)=ce^{i\alpha_{k}n+zt}=x_{n}(t)-{\bar{x}}_{n}\left(t\right),\;y_{n}\left(t\right)\to 0,\;\alpha_{k}=\frac{2\pi k}{N}$$

where c is a constant and $\alpha_{k}=\frac{2\pi k}{N}(k=0,1,\ldots ,N-1)$

Taking the second derivative of both sides of Equation (14), we obtain

(15) $${\ddot{y}}_{n}\left(t+t_{d}\right)={\ddot{x}}_{n}\left(t+t_{d}\right)-{\left({\bar{x\;}}_{n}\left(t+t_{d}\right)\right)}^{\prime \prime }={\ddot{x}}_{n}\left(t+t_{d}\right)=\frac{dv_{n}\left(t+t_{d}\right)}{dt}$$

Based on Equation (2), we rewrite Equation (15) as

(16) $${\ddot{y}}_{n}\left(t+t_{d}\right)=f_{n}(s_{n}(t),\;v_{n}(t),\;\Delta v_{n}(t),\;s_{n+1}(t),\;\Delta v_{n+1}(t))$$

By linearizing Equation (16), we can derive the following equation:

(17) $${\ddot{y}}_{n}\left(t+t_{d}\right)=f_{s_{n}}\left(y_{n-1}\left(t\right)-y_{n}\left(t\right)\right)+f_{v_{n}}{\dot{y}}_{n}\left(t\right)+f_{\Delta v_{n}}\left({\dot{y}}_{n-1}\left(t\right)-{\dot{y}}_{n}\left(t\right)\right)\;+f_{s_{n+1}}\left(y_{n}\left(t\right)-y_{n+1}\left(t\right)\right)+f_{\Delta v_{n+1}}\left({\dot{y}}_{n}\left(t\right)-{\dot{y}}_{n+1}\left(t\right)\right))$$

where $f_{s_{n}}={\left.\frac{\partial f_{n}}{\partial s_{n}}\right|}_{\left(\bar{v},\;\bar{s}\right)}>0$, $f_{v_{n}}={\left.\frac{\partial f_{n}}{\partial v_{n}}\right|}_{\left(\bar{v},\;\bar{s}\right)}<0$, $f_{\Delta v_{n}}={\left.\frac{\partial f_{n}}{\partial {\Delta v}_{n}}\right|}_{\left(\bar{v},\;\bar{s}\right)}<0$, $f_{s_{n+1}}={\left.\frac{\partial f_{n}}{\partial s_{n+1}}\right|}_{\left(\bar{v},\;\bar{s}\right)}$, and $f_{\Delta v_{n+1}}={\left.\frac{\partial f_{n}}{\partial {\Delta v}_{n+1}}\right|}_{\left(\bar{v},\;\bar{s}\right)}$.

We rewrite Equation (17) and substitute $y_{n}\left(t\right)=ce^{i\alpha_{k}n+zt}$ and ${\dot{y}}_{n}\left(t\right)=zce^{i\alpha_{k}n+zt}$ into Equation (17). Simplifying the resulting equation, we can obtain

(18) $$\left(e^{t_{d}z}-1\right)[ze^{t_{d}z}-f_{v_{n}}+\left(e^{-ia_{k}}-1\right){(f}_{∆v_{n}}+{e^{ia_{k}}f}_{∆v_{n+1}})]\;=t_{d}\cdot \left(e^{-ia_{k}}-1\right)(f_{s_{n}}+{e^{ia_{k}}f}_{s_{n+1}})$$

We expand z in a power series solution, where $z=z_{1}\left(ia_{k}\right)+z_{2}{\left(ia_{k}\right)}^{2}+\cdots$ and $e^{t_{d}z}=1+t_{d}z+\frac{t_{d}^{2}z^{2}}{2}+\cdots$. We can insert this solution into Equation (18) to derive the first-order and second-order terms of coefficients in expression of z, given, respectively, the following:

(19) $$z_{1}=\frac{f_{s_{n}}+f_{s_{n+1}}}{f_{v_{n}}}$$

(20) $$z_{2}=\frac{z_{1}^{2}-z_{1}(f_{\Delta v_{n}}+f_{\Delta v_{n+1}})-\frac{1}{2}(f_{s_{n}}-f_{s_{n+1}})}{f_{v_{n}}}-\frac{1}{2}(z_{1}^{2}t_{d})$$

The platoon is string stable if $z_{2}>0$. The string stability condition is derived as follows, assuming no time delays ($t_{d}=0)$:

(21) $${\;{(f}_{s})}^{2}-\frac{{{(f}_{v_{n}})}^{2}}{2}\left(f_{s_{n}}-f_{s_{n+1}}\right)-f_{s}f_{v_{n}}f_{∆v}<0$$

where $f_{s}=f_{s_{n}}+f_{s_{n+1}},f_{∆v}=f_{\Delta v_{n}}+f_{\Delta v_{n+1}}$

If $f_{s_{n+1}}=0$ and $f_{∆v_{n+1}}=0$, the equation then simplifies to $f_{s_{n}}-\frac{{{(f}_{n}^{v})}^{2}\;}{2}-{f_{v_{n}}f}_{\Delta v_{n}}<0,$ which matches the string stability condition of the traditional predecessor following scheme [40,49,50].

This section first derives the stability condition of the spacing bidirectional control model based on Equation (21) when $f_{\Delta v_{n+1}}=0$ and $f_{s_{n+1}}\neq 0$. The platoon is stable if $\eta^{s}<0,$ which is formulated in Equation (22),

(22) $$\eta^{s}={\;{(f}_{s})}^{2}-\frac{{{(f}_{v_{n}})}^{2}}{2}\left(f_{s_{n}}-f_{s_{n+1}}\right)-f_{s}f_{v_{n}}f_{\Delta v_{n}}$$

It should be noted that in the spacing bidirectional control model, spacing information $s_{n+1}$ from the following vehicle is usually coupled with spacing information $s_{n}$ from the preceding vehicle as a stimulative term, $w\left(s_{n+1}\right)-w\left(s_{n}\right)$. Therefore, $f_{n}^{s}$ is composed of two parts, denoted as $f_{n}^{s1}$ and $f_{n}^{s2}$, where $f_{n}^{s1}$ represents the original part in Equation (1) and $f_{n}^{s2}={-f}_{n+1}^{s}$ represent the part in a stimulative term as shown in the equation below:

(23) $$f_{s_{n}}=f_{s_{n}}^{1}+f_{s_{n}}^{2}$$

where $f_{s_{n}}^{2}=-f_{s_{n+1}}$. 

Then, the stability condition of the spacing bidirectional control model can be rewritten as shown in Equation (24),

(24) $${{\eta^{s}=(f}_{n}^{s1})}^{2}-\frac{{{(f}_{v_{n}})}^{2}}{2}\left(f_{s_{n}}^{1}-2f_{s_{n+1}}\right)-f_{s_{n}}^{1}f_{v_{n}}f_{\Delta v_{n}}$$

Taking the partial derivative of Equation (24) with respect to $f_{n+1}^{s}$ yields Equation (25) as follows:

(25) $$\frac{{\partial \eta }^{s}}{\partial f_{s_{n+1}}}={{(f}_{v_{n}})}^{2}$$

Since $\frac{{\partial \eta }^{s}}{\partial f_{s_{n+1}}}>0$, it can be observed that when $f_{s_{n+1}}<0$ (IP), $\eta^{s}$ decreases, indicating an improvement in string stability. On the contrary, when $f_{s_{n+1}}>0$ (OP), $\eta^{s}$ increases, leading to worse stability.

In this section, we derive the stability condition of the velocity difference bidirectional control model based on Equation (21) when $f_{\Delta v_{n+1}}\neq 0$ and $f_{s_{n+1}}=0$. The platoon is stable if $\eta^{\Delta v}<0$, which is formulated in Equation (26),

(26) $${\eta^{\Delta v}=(f_{s_{n}})}^{2}-\frac{{{(f}_{v_{n}})}^{2}}{2}f_{s_{n}}-f_{s_{n}}f_{v_{n}}f_{∆v}$$

Taking the partial derivative of Equation (26) with respect to $f_{\Delta v_{n+1}}$ yields Equation (27),

(27) $$\frac{\partial \eta^{∆v}}{\partial f_{\Delta v_{n+1}}}=-f_{s_{n}}f_{v_{n}}$$

where $f_{s_{n}}>0$ and $f_{v_{n}}<0$. 

Since $\frac{\partial \eta^{∆v}}{\partial f_{\Delta v_{n+1}}}>0$, it can be observed that when $f_{\Delta v_{n+1}}<0$ (OP), $\eta^{\Delta v}$ decreases, resulting in improved stability. On the contrary, when $f_{\Delta v_{n+1}}>0$ (IP), $\eta^{\Delta v}$ increases, leading to worse stability.

### 3.6. Tradeoff between Platoon Safety and String Stability

Section 3.4 and Section 3.5 investigate the collision risk and string stability of the spacing and velocity difference bidirectional control models. In this section, we synthesize the findings and discuss the tradeoff between platoon safety and stability.

First, we direct our attention to the spacing bidirectional control model. The analyses conducted in Section 3.4 and Section 3.5 indicate that a bidirectional control model exhibiting the in-phase (IP) effect has the potential to reduce the risk of rear-end collisions and enhance string stability. Conversely, a model featuring the opposite-phase (OP) effect can increase the likelihood of rear-end collisions and worsen instability. These observations are summarized in Table 2, highlighting that incorporating spacing information with the IP effect can effectively improve both platoon stability and safety.

Next, our attention turns to the evaluation of the velocity difference bidirectional control model. The analysis conducted in Section 3.4 and Section 3.5 reveals notable observations: the IP effect can decrease the risk of rear-end collisions, albeit at the cost of worsening stability. Conversely, the OP effect can increase the risk of rear-end collisions but has the potential to improve string stability. Table 3 summarizes these findings, emphasizing that the utilization of velocity difference information can only enhance either safety or stability, but not both simultaneously.

## 4. Analytical Verification on Specific Car-Following Models

The objective of this section is to provide detailed analytical validation for the theoretical findings presented in the previous sections. This is accomplished by examining the safety and stability of two distinct car-following models: one linear model (i.e., Helly’s model) and one non-linear model (i.e., the IDM). Both of them are prominent in modeling CAV behaviors [33,51,52,53]. Incorporating both a linear and a non-linear model allows analytical verification to provide comprehensive insights.

### 4.1. Linear Car-following Model

Car-following models have been developed for more than half a century, and numerous models have been proposed to model the longitudinal behaviors of vehicles. Among them, Helly’s linear car-following model has been widely applied to describe CAV behavior [54] due to its simple and intuitive feature [40]. This research adopts Helly’s car-following model to model the linear following behavior of CAVs and bidirectional information flow topology effects. The formulation of the Helly’s model is denoted as below [55]:

(28) $${\dot{v}}_{n}\left(t\right)=\lambda_{x}\left(s_{n}\left(t\right)-\tau v_{n}\left(t\right)-s_{0}\right)-\lambda_{v}∆v_{n}(t)$$

where $\lambda_{v}\;$represents the sensitivity to velocity difference between target vehicle and preceding vehicle, $\lambda_{x}$ represents the sensitivity to spacing between target vehicle and preceding vehicle, $s_{0}$ is the minimum distance allowed as a safety gap, and τ represents reaction time. Note that $\tau v_{n}\left(t\right)+s_{0}$ indicates the desired space gap. In this model, the acceleration of a vehicle presents a linear relationship with deviation from spacing and velocity difference between two successive vehicles.

Incorporating the information from the nearest following vehicle, the bidirectional Helly’s model can be expressed as:

(29) $${\dot{v}}_{n}\left(t\right)=\lambda_{x}\left(s_{n}\left(t\right)-\tau v_{n}\left(t\right)-s_{0}\right)-\lambda_{v}∆v_{n}\left(t\right)\;\;\;\;\;\;\;\;\;\;+\gamma_{x}(s_{n+1}\left(t\right){-s}_{n}\left(t\right))+\gamma_{v}∆v_{n+1}\left(t\right)$$

where $s_{n+1},\;\Delta v_{n+1}$ represent the spacing and velocity difference information from the following vehicle, respectively. Difference $s_{n+1}\left(t\right){-s}_{n}\left(t\right)$ represents the spacing stimulus. $\gamma_{x}$ represents sensitivity to spacing, and $\gamma_{v}\;$represents sensitivity to velocity difference. By setting $\gamma_{v}=0$ and $\gamma_{x}=0$, the spacing and the velocity difference bidirectional Helly’s model can be obtained, respectively.

We can obtain the analytic expressions of the partial differential equations for the bidirectional Helly’s model, as shown in the equations below:

(30) $$f_{s_{n}}^{1}=\lambda_{x},\;{\;f}_{s_{n}}^{2}=-\gamma_{x},\;{\;f}_{v_{n}}=-\lambda_{x}\tau ,\;{\;f}_{∆v_{n}}=-\lambda_{v},\;\;\;\;\;\;\;\;\;\;\;f_{s_{n+1}}=\gamma_{x},\;f_{∆v_{n+1}}=\gamma_{v}$$

For the spacing bidirectional Helly’s model, by applying the partial differential equations to Equation (10), we obtain Equation (31).

(31) $${\left.\frac{\partial TTC_{n+1}^{s}\left(t+\Delta t\right)}{\partial f_{s_{n+1}}}\right|}_{\left(\bar{v},\;\bar{s}\right)}=\frac{(\varphi_{n+1}\left(t+\Delta t\right)+s_{e})\varphi_{n+1}\left(t\right)\Delta t}{{{(\mu }_{n+1}\left(t+\Delta t\right)-\gamma_{x}\varphi_{n+1}\left(t\right)\Delta t)}^{2}}$$

Since $\frac{\partial TTC_{n+1}^{s}\left(t+\Delta t\right)}{\partial f_{s_{n+1}}}<0$, it can be observed that when $f_{s_{n+1}}<0$ (IP), $TTC_{n+1}\left(t+\Delta t\right)$ increases, resulting in lower collision risk. On the contrary, when $f_{s_{n+1}}>0$ (OP), $TTC_{n+1}\left(t+\Delta t\right)$ decreases, leading to higher collision risk.

For the velocity difference bidirectional Helly’s model, by applying the partial differential equations to Equation (12), we obtain Equation (32).

(32) $${\left.\frac{\partial TTC_{n+1}^{\Delta v}\left(t+\Delta t\right)}{\partial f_{\Delta v_{n+1}}}\right|}_{\left(\bar{v},\;\bar{s}\right)}=\frac{(\varphi_{n+1}\left(t+\Delta t\right)+s_{e})\mu_{n+1}(t)\Delta t}{{{(\mu }_{n+1}\left(t+\Delta t\right)-\gamma_{v}\mu_{n+1}(t)\Delta t)}^{2}}$$

Since $\frac{\partial TTC_{n+1}^{\Delta v}\left(t+\Delta t\right)}{\partial f_{\Delta v_{n+1}}}>0$, it can be observed that when $f_{\Delta v_{n+1}}>0$ (IP), $TTC_{n+1}^{\Delta v}\left(t+\Delta t\right)$ increases, implying lower collision risk. On the contrary, when $f_{\Delta v_{n+1}}<0$ (OP), $TTC_{n+1}^{\Delta v}\left(t+\Delta t\right)$ decreases, leading to higher collision risk. The above rear-end collision risk analyses on Helly’s model demonstrate the findings presented in Table 2 and Table 3.

**Figure 4:**
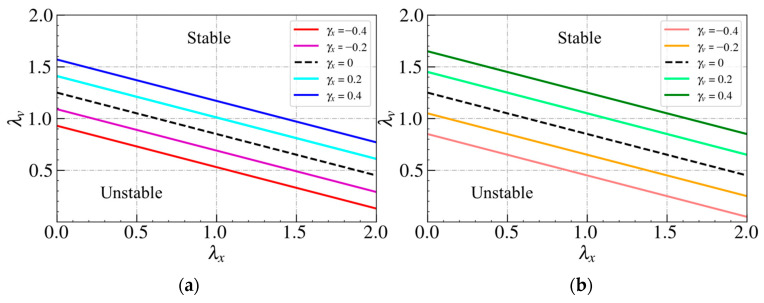
Stability transition curves for (**a**) spacing, (**b**) velocity difference bidirectional Helly’s model.

We now proceed to derive the stability condition of the bidirectional Helly’s model. Applying the partial differential equations to Equations (22) and (26), we can obtain the stability transition curves for both the spacing and velocity difference bidirectional models from the neutral stability criterion. Figure 4 presents the stability transition curves for both models. Figure 4a shows the stability transition curve of the spacing bidirectional Helly’s model, while Figure 4b shows the stability transition curve of the velocity difference bidirectional Helly’s model. The traffic flow is considered stable when the equilibrium space gap lies above the stability line. The black dashed line represents the phase transition curve of Helly’s model when $\gamma_{x}=0$ and $\gamma_{v}=0$.

Figure 4a for the spacing bidirectional Helly’s model indicates that the stability region expands when $\gamma_{x}<0$ (IP). On the contrary, when $\gamma_{x}>0$ (OP), the stability region shrinks. Figure 4b for the velocity difference bidirectional Helly’s model reveals that when $\gamma_{v}>0$ (IP), the stability region shrinks, and the stability region expands when $\gamma_{v}<0$ (OP). The above string stability analyses on Helly’s model demonstrate the findings presented in Table 2 and Table 3.

### 4.2. Non-Linear Car-following Model

The research further employs the Intelligent Driver Model (IDM) car-following model, proposed by Treiber et al. [12], to describe the non-linear following behavior of CAVs due to several of its advantages. First, the IDM is a multi-regime model, which captures the dynamics of different traffic congestion levels more realistically than other models [33]. Second, it provides collision-free behavior and smooth traffic flow [56]. Third, it is well-accepted to model connected automated vehicles’ longitudinal dynamics [57]. 

In the IDM model, the acceleration of vehicle n at time *t* is determined by its current velocity $v_{n}$, headway $s_{n}$, and velocity difference $\Delta v_{n}$ to the preceding vehicle, which can be expressed as

(33) $$\dot{v_{n}}\left(t\right)=a\left(1-{\left(\frac{v_{n}(t)}{v_{0}}\right)}^{\delta }-{\left(\frac{s^{*}\left(v_{n}(t),\;∆v_{n}(t)\right)}{s_{n}(t)}\right)}^{2}\right)\;s^{*}\left(v_{n}(t),\;∆v_{n}(t)\right)=s_{0}+v_{n}(t)T+\frac{v_{n}(t)\cdot ∆v_{n}(t)}{2\sqrt{ab}}$$

where $\dot{v_{n}}\left(t\right)$ and $v_{n}$ denote the acceleration and speed of vehicle *n* at time *t*; a and b denote the maximum acceleration and deceleration of the vehicle, respectively; $v_{0}$ denotes the free-flow speed; δ is the acceleration exponent parameter; $s_{0}$ represents the minimum bumper-to-bumper gap in traffic jam states; *T* is the desired time gap; $s_{n}$ denotes the net distance, $s_{n}=x_{n}-x_{n-1}-l,$ between vehicle *n* and its preceding vehicle *n* − 1, where l is vehicle length and $x_{n}$ denotes the position of vehicle *n* at time *t*. $∆v_{n}$ denotes the velocity difference between vehicle *n* and its preceding vehicle *n* − 1.

The research adapts the bidirectional distance balanced model (BDBM) proposed in [26] and further modifies it by incorporating velocity difference information from the nearest following vehicle. The structure of the new bidirectional IDM model is expressed as follows:

(34) $$\dot{v_{n}}\left(t\right)=a\left(1-{\left(\frac{v_{n}(t)}{v_{0}}\right)}^{\delta }-{\left(\frac{s^{*}\left(v_{n}(t),\;∆v_{n}(t)\right)}{s_{n}(t)}\right)}^{2}\right)\;s^{*}\left(v_{n}\left(t\right),\;∆v_{n}\left(t\right)\right)=s_{0}+v_{n}\left(t\right)T+\frac{v_{n}\left(t\right)\cdot ∆v_{n}\left(t\right)}{2\sqrt{ab}}\;+\gamma_{x}\left(s_{n+1,t}(t)-s_{n,t}(t)\right)+\gamma_{v}∆v_{n+1}(t)$$

where $s_{n+1}$ and $\Delta v_{n+1}$ denote the spacing and velocity difference information received from the following vehicle, respectively. $\gamma_{x}$ represents the sensitivity to spacing and $\gamma_{v}\;$represents the sensitivity to velocity difference. By setting $\gamma_{v}=0$ and $\gamma_{x}=0$, the spacing and the velocity difference bidirectional IDM model can be obtained, respectively.

We can obtain the analytic expressions of the partial differential equations for the bidirectional IDM model, as shown in the equations below:

(35) $$f_{s_{n}}^{1}=\frac{2a{s^{*}}^{2}}{s^{3}},\;{\;f}_{s_{n}}^{2}=\frac{2as^{*}\gamma_{x}}{s^{2}},\;{\;f}_{v_{n}}=-\frac{4av_{e}^{3}}{v_{o}^{4}}-\frac{2a{Ts}^{*}}{s^{2}},\;{\begin{array}{c} f \end{array}}_{∆v_{n}}=-\frac{\sqrt{a}{vs}^{*}}{s^{2}\sqrt{b}},\;f_{s_{n+1}}=-\frac{2as^{*}\gamma_{x}}{s^{2}}\;,\;f_{∆v_{n+1}}=-\frac{2as^{*}\gamma_{v}}{s^{2}}$$

For the spacing bidirectional IDM, by applying the partial differential equations in Equation (35) to Equation (10), we obtain Equation (36).

(36) $${\left.\frac{\partial TTC_{n+1}^{s}\left(t+\Delta t\right)}{\partial f_{s_{n+1}}}\right|}_{\left(\bar{v},\;\bar{s}\right)}=\frac{(\varphi_{n+1}\left(t+\Delta t\right)+s_{e})\varphi_{n+1}\left(t\right)\Delta t}{{{(\mu }_{n+1}\left(t+\Delta t\right)+\frac{2as^{*}\gamma_{x}}{s^{2}}\varphi_{n+1}\left(t\right)\Delta t)}^{2}}$$

Since $\frac{\partial TTC_{n+1}^{s}\left(t+\Delta t\right)}{\partial f_{s_{n+1}}}<0$, it can be observed that when $\gamma_{x}>0$ and $f_{s_{n+1}}<0$ (IP), $TTC_{n+1}\left(t+\Delta t\right)$ increases, resulting in lower collision risk. On the contrary, when $\gamma_{x}<0$ and $f_{s_{n+1}}>0$ (OP), $TTC_{n+1}\left(t+\Delta t\right)$ decreases, leading to higher collision risk.

For the velocity difference bidirectional IDM, by applying the partial differential equations in Equation (35) to Equation (12), we can obtain

(37) $${\left.\frac{\partial TTC_{n+1}^{\Delta v}\left(t+\Delta t\right)}{\partial f_{\Delta v_{n+1}}}\right|}_{\left(\bar{v},\;\bar{s}\right)}=\frac{(\varphi_{n+1}\left(t+\Delta t\right)+s_{e})\mu_{n+1}(t)\Delta t}{{{(\mu }_{n+1}\left(t+\Delta t\right)+\frac{2as^{*}\gamma_{v}}{s^{2}}\mu_{n+1}(t)\Delta t)}^{2}}$$

Since $\frac{\partial TTC_{n+1}^{\Delta v}\left(t+\Delta t\right)}{\partial f_{\Delta v_{n+1}}}>0$, it can be observed that when $\gamma_{v}<0$ and $f_{\Delta v_{n+1}}>0$ (IP), $TTC_{n+1}^{\Delta v}\left(t+\Delta t\right)$ increases, resulting in lower collision risk. On the contrary, when $\gamma_{v}>0$ and $f_{\Delta v_{n+1}}<0$ (OP), $TTC_{n+1}^{\Delta v}\left(t+\Delta t\right)$ decreases, leading to higher collision risk. The above rear-end collision risk analyses on the bidirectional IDM model validate the findings presented in Table 2 and Table 3.

We now derive the stability condition of the bidirectional IDM model, using the parameters in [46] listed in Table 4. By applying the partial differential equations in Equation (35) to Equations (22) and (26), we can obtain the stability transition curves for both the spacing and velocity difference bidirectional IDM models from the neutral stability criterion.

**Figure 5:**
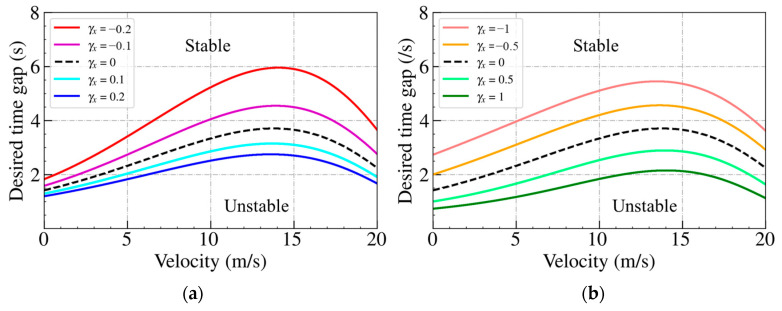
Stability transition curve for (**a**) spacing, (**b**) velocity difference bidirectional IDM model.

Figure 5 illustrates the stability transition curves for both models. The figure to the left shows the stability transition curve of the spacing bidirectional IDM model, while the figure to the right shows the stability transition curve of the velocity difference bidirectional IDM model. The traffic flow is considered stable when the equilibrium space gap lies above the stability line. The black dashed line represents the phase transition curve of the IDM model when $\gamma_{x}=0$ and $\gamma_{v}=0$.

The figure to the left for the spacing bidirectional IDM model indicates that the stability region expands when $\gamma_{x}>0$ (IP). On the contrary, the stability region shrinks when $\gamma_{x}<0$ (OP). The figure to the right for the velocity difference bidirectional IDM model reveals that the stability region shrinks when $\gamma_{v}<0$ (IP) and the stability region expands when $\gamma_{v}>0$ (OP). The above string stability analyses on the bidirectional IDM model validate the findings presented in Table 2 and Table 3.

## 5. Numerical Verification

In this section, we perform numerical experiments using the bidirectional Helly’s model and the bidirectional IDM model to validate the analytical findings presented in Section 3 and Section 4. The simulations are initialized as follows: A platoon consisting of 20 vehicles operates on a single lane with an open boundary condition. All vehicles have an identical initial velocity of 15 m/s and are spaced equidistantly. To investigate the impact of back-looking information on vehicle dynamics, perturbation is introduced to the 10th vehicle in the platoon. The 10th vehicle is programmed to follow a trapezoidal-type speed profile, simulating a typical congested traffic scenario characterized by sudden acceleration and deceleration.

### 5.1. Numerical Investigation on Helly’s Model with Bidirectional Information

Numerical experiments using the spacing bidirectional Helly’s model are first carried out. The values of model parameters are from [58] as summarized in Table 5.

**Figure 6:**
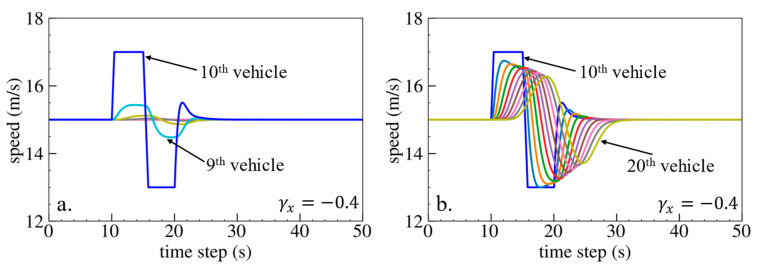
Vehicle speed profile under the bidirectional Helly’s model when $\gamma_{x}=-0.4:$ (**a**) 1st–10th vehicles, (**b**) 10th–20th vehicles.

**Figure 7:**
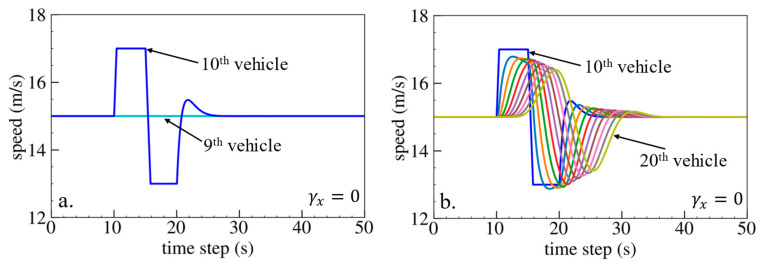
Vehicle speed profile under the bidirectional Helly’s model when $\gamma_{x}=0:$ (**a**) 1st–10th vehicles, (**b**) 10th–20th vehicles.

**Figure 8:**
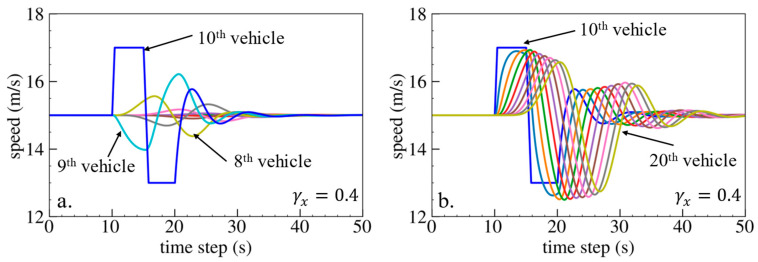
Vehicle speed profile under the bidirectional Helly’s model when $\gamma_{x}=0.4:$ (**a**) 1st–10th vehicles, (**b**) 10th–20th vehicles.

**Figure 9:**
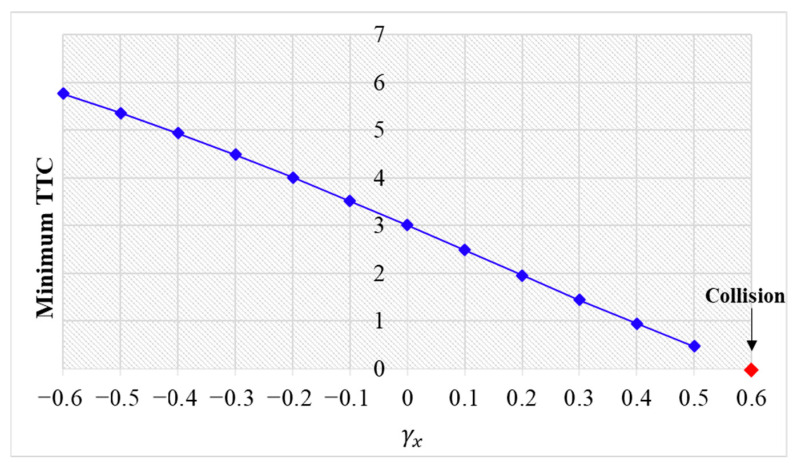
Minimum TTC variation with respect to $\gamma_{x}$ in the bidirectional Helly’s model.

The simulation results are shown below. Figure 6, Figure 7 and Figure 8 show vehicle speed profiles when $\gamma_{x}$= −0.4, 0 and 0.4, respectively. Note that when $\gamma_{x}=0$, the model degenerates to Helly’s model. In each figure, Figure 6a, Figure 7a and Figure 8a show the speed profile of the perturbed vehicle and its preceding vehicles, demonstrating how back-looking information impacts downstream vehicle dynamics, especially in terms of safety. Figure 6b, Figure 7b and Figure 8b show the speed profile of the perturbed vehicle and its following vehicles, demonstrating how back-looking information impacts upstream vehicles dynamics, especially in terms of string stability. Figure 9 illustrates the minimum TTC value between the perturbed vehicle and its nearest preceding vehicle.

When $\gamma_{x}=-0.4$ and $f_{n+1}^{s}<0$, based on the analysis in Table 2, spacing information results in the IP effect. As shown in Figure 6a, when the perturbed 10th vehicle undergoes abrupt acceleration and deceleration, the nearest preceding 9th vehicle also accelerates and decelerates. In Figure 9, a larger minimum TTC value is observed when $\gamma_{x}=-0.4$ compared to the value when $\gamma_{x}=0$, indicating lower collision risk. Figure 6b demonstrates that the following vehicles smoothly converge to steady speed without significant oscillations. These results validate that incorporating spacing information with the IP effect can reduce collision risk and improve platoon stability.

When $\gamma_{x}=0.4$ and $f_{n+1}^{s}>0$, spacing information results in the OP effect. As shown in Figure 8a, when the perturbed 10th vehicle undergoes abrupt acceleration and deceleration, the nearest preceding 9th vehicle reacts by decelerating and then accelerating adversely. The speed variation of the ninth vehicle also affects the eighth vehicle, resulting in adverse behavior with respect to the ninth vehicle. In Figure 9, a smaller minimum TTC value is observed when $\gamma_{x}=0.4$, indicating higher collision risk. Figure 8b shows that the speed fluctuations are enlarged upstream in the platoon due to string instability. These results validate that incorporating spacing information with the OP effect can increase collision risk and worsen platoon stability.

Figure 9 demonstrates that an increase in $\gamma_{x}$ leads to a smaller minimum TTC value, indicating higher collision risk. When $\gamma_{x}$ approaches 0.6, the perturbed 10th vehicle collides with the 9th vehicle due to the OP effect.

**Figure 10:**
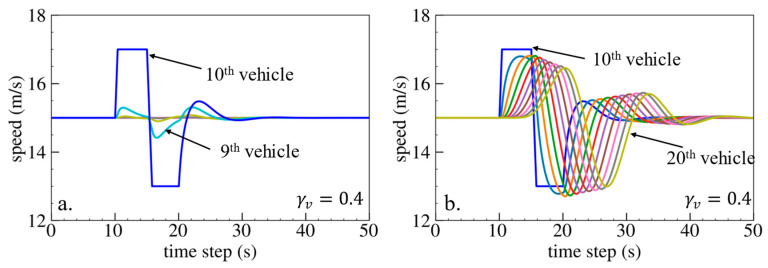
Vehicle speed profile under the bidirectional Helly’s model when $\gamma_{v}=0.4:$ (**a**) 1st–10th vehicles, (**b**) 10th–20th vehicles.

**Figure 11:**
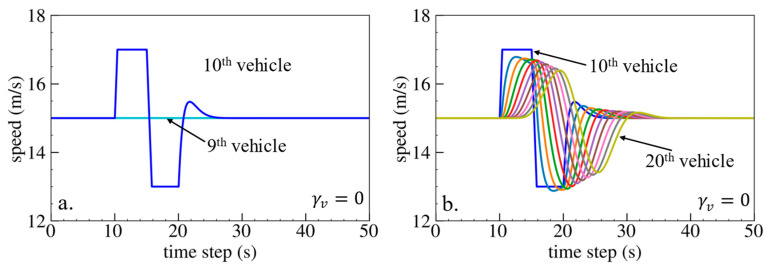
Vehicle speed profile under the bidirectional Helly’s model when $\gamma_{v}=0:$ (**a**) 1st–10th vehicles, (**b**) 10th–20th vehicles.

**Figure 12:**
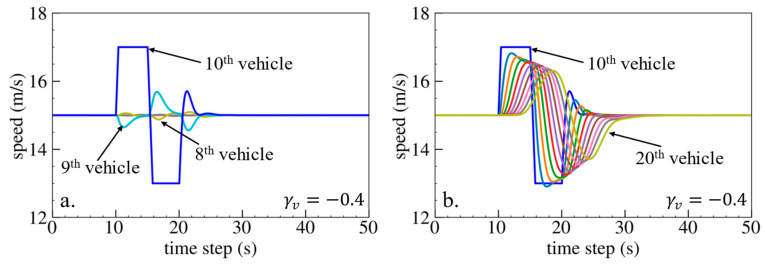
Vehicle speed profile under the bidirectional Helly’s model when $\gamma_{v}=-0.4:$ (**a**) 1st–10th vehicles, (**b**) 10th–20th vehicles.

**Figure 13:**
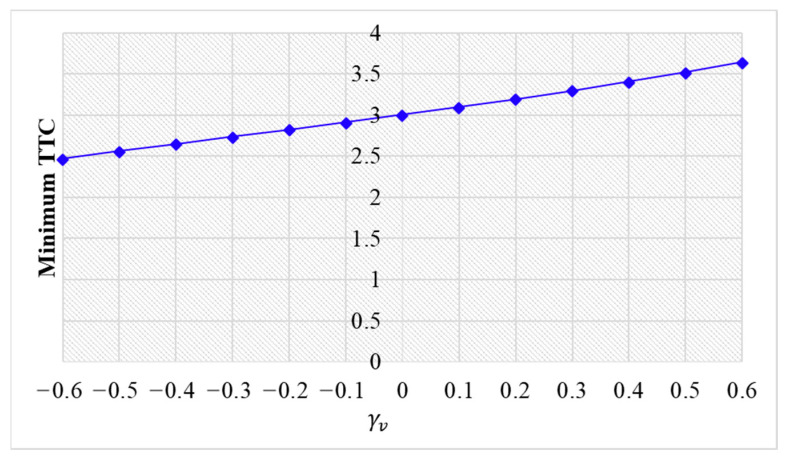
Minimum TTC variation with respect to $\gamma_{v}$ in the bidirectional Helly’s model.

Numerical experiments using the velocity difference bidirectional Helly’s model are then carried out. Figure 10, Figure 11 and Figure 12 show the vehicle speed profiles when $\gamma_{v}$= 0.4, 0 and −0.4, respectively. Figure 13 illustrates the minimum TTC value between the perturbed vehicle and its nearest preceding vehicle.

When $\gamma_{v}=0.4$ and $f_{n+1}^{∆v}>0$, the velocity difference information results in the IP effect. As shown in Figure 10a, when the perturbed 10th vehicle undergoes abrupt acceleration and deceleration, the nearest preceding 9th vehicle also accelerates and decelerates. The minimum TTC value increases in Figure 13, indicating lower collision risk. In Figure 10b, the speed fluctuations are enlarged upstream in the platoon due to string instability. These results validate that incorporating velocity information with the IP effect can reduce collision risk but worsen platoon stability.

When $\gamma_{v}=-0.4$ and $f_{n+1}^{∆v}<0$, velocity difference information results in the OP effect. As shown in Figure 12a, when the perturbed 10th vehicle undergoes abrupt acceleration and deceleration, the nearest preceding 9th vehicle reacts by decelerating and then accelerating adversely. In Figure 13, a smaller minimum TTC value is observed, indicating higher collision risk. Figure 12b demonstrates that the following vehicles smoothly converge to steady speed without significant oscillation. These results confirm that incorporating velocity information with the OP effect can improve platoon stability but increase collision risk.

Figure 13 illustrates that as parameter $\gamma_{v}$ increases, there is a reduction in the minimum TTC value. This trend suggests a decreased risk of collision. Moreover, it is observed that no collisions occur across the entire range of $\gamma_{v}$ values examined.

### 5.2. Numerical Investigation on the IDM with Bidirectional Information

**Figure 14:**
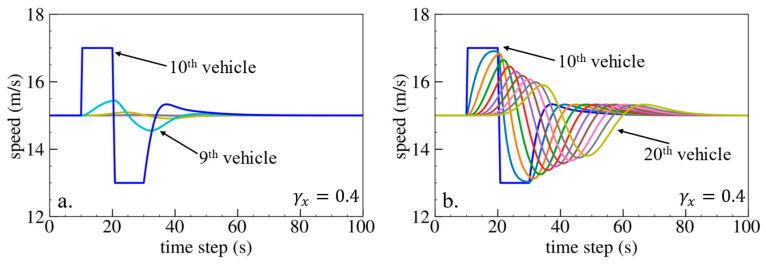
Vehicle speed profile under the bidirectional IDM when $\gamma_{x}=0.4:$ (**a**) 1st–10th vehicles, (**b**) 10th–20th vehicles.

**Figure 15:**
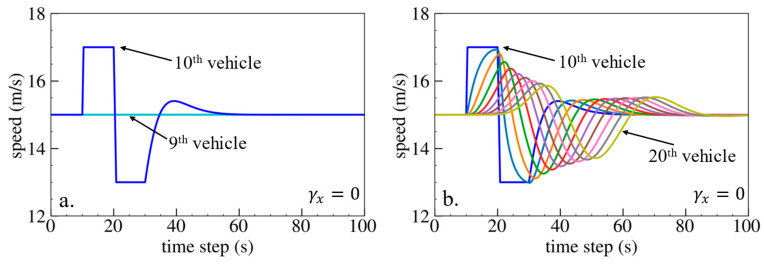
Vehicle speed profile under the bidirectional IDM when $\gamma_{x}=0:$ (**a**) 1st–10th vehicles, (**b**) 10th–20th vehicles.

**Figure 16:**
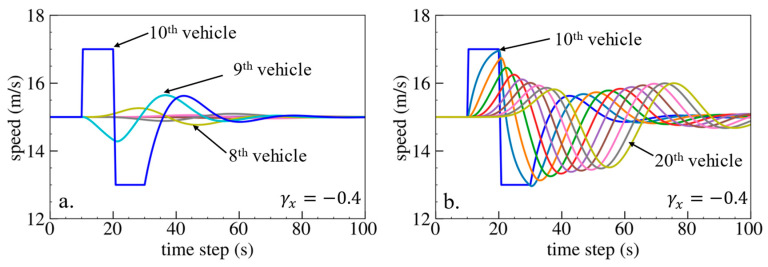
Vehicle speed profile under the bidirectional IDM when $\gamma_{x}=-0.4:$ (**a**) 1st–10th vehicles, (**b**) 10th–20th vehicles.

**Figure 17:**
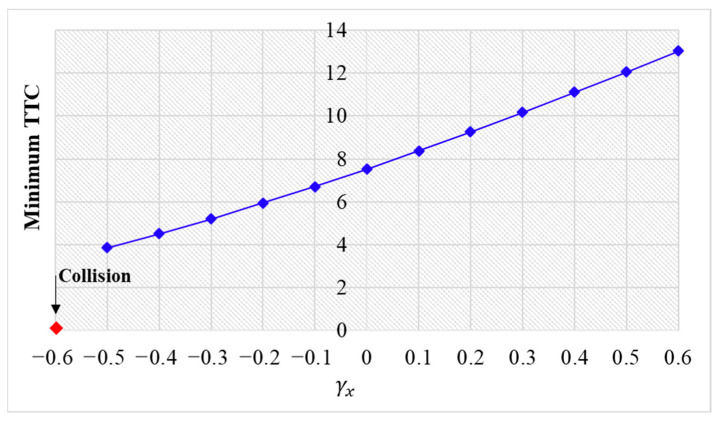
Minimum TTC variation with respect to $\gamma_{x}$ in the bidirectional IDM.

The spacing bidirectional IDM is subject to numerical experiments to validate the findings. The parameters used in the experiments are identical to those in Table 5. Figure 14, Figure 15 and Figure 16 demonstrate the vehicle speed profiles when $\gamma_{x}$= 0.4, 0 and −0.4, respectively. Figure 17 depicts the minimum TTC value among 1st–10th vehicles. These simulation outcomes are consistent with the previous simulation results obtained using Helly’s model. Specifically, utilizing spacing information with the IP effect can effectively reduce collision risk and enhance platoon stability. On the contrary, incorporating spacing information with the OP effect can increase collision risk and worsen platoon stability.

**Figure 18:**
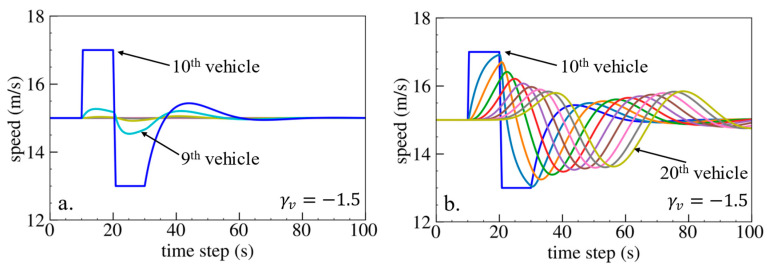
Vehicle speed profile under the bidirectional IDM when $\gamma_{v}=-1.5:$ (**a**) 1st–10th vehicles, (**b**) 10th–20th vehicles.

**Figure 19:**
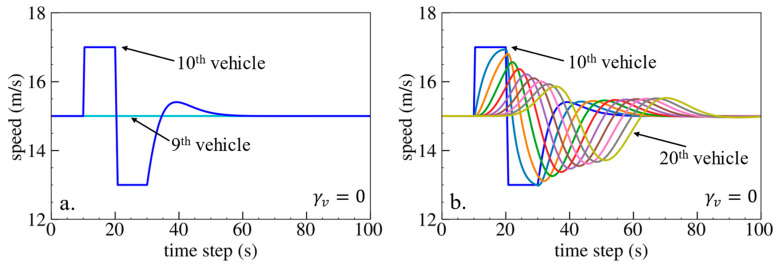
Vehicle speed profile under the bidirectional IDM when $\gamma_{v}=0:$ (**a**) 1st–10th vehicles, (**b**) 10th–20th vehicles.

**Figure 20:**
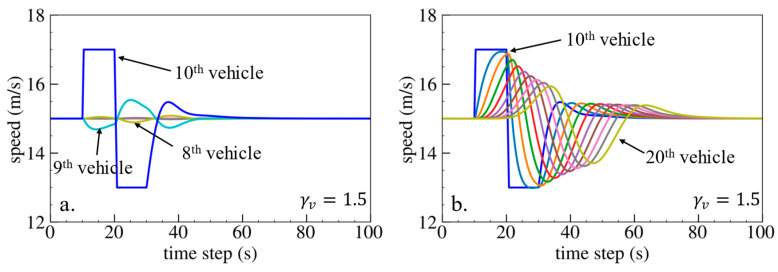
Vehicle speed profile under the bidirectional IDM when $\gamma_{v}=1.5:$ (**a**) 1st–10th vehicles, (**b**) 10th–20th vehicles.

**Figure 21:**
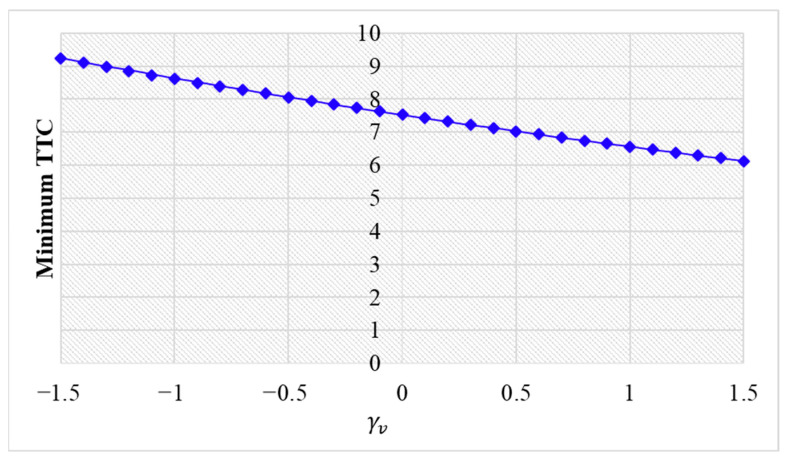
Minimum TTC variation with respect to $\gamma_{v}$ in the bidirectional IDM.

Numerical experiments are further conducted using the velocity difference bidirectional IDM model. Figure 18, Figure 19 and Figure 20 depict the vehicle speed profiles when $\gamma_{v}$= −1.5, 0 and 1.5, respectively. Figure 21 displays the minimum TTC value. The simulation results are in line with the previous findings obtained using Helly’s model. Specifically, using velocity difference information with the IP effect can reduce collision risk but worsen platoon stability, whereas velocity difference information with the OP effect can improve platoon stability but increase collision risk.

In conclusion, the numerical analyses conducted on both linear and nonlinear models verify the findings presented in Table 3 and Table 4. The use of spacing information can enhance both platoon string stability and safety. However, the use of velocity difference information can only enhance either safety or stability.

## 6. Conclusions

This paper introduces a novel framework based on the concept of phase shift to investigate the influence of bidirectional information flow topology on platoons in terms of both stability and safety. This research sheds light on the effects of phase shift, particularly focusing on two specific cases: the in-phase (IP) effect and the opposite-phase (OP) effect. The IP effect contributes to enhanced platoon safety, while the OP effect significantly increases the risk of rear-end collisions. By employing the proposed framework, the research investigates the impact of different types of dynamics information of vehicles on platoons. Theoretical analyses pertaining to string stability and rear-end collision risk reveal that the integration of diverse information into the models does not universally yield benefits. Specifically, incorporating spacing information can concurrently improve both platoon safety and stability. However, the integration of velocity difference information can only enhance either safety or stability, but not both simultaneously. To validate these theoretical analyses, numerical experiments are conducted on both linear and non-linear car-following models, with simulation results confirming theoretical analyses. 

There are several potential directions for future research in this field. While this research focuses on uncovering the effects of phase shift on CAV platoons, it primarily examines the IP and OP effects which represent two extreme cases of phase shift. Other communication factors, such as delay and packet loss, might result in different phase shift effects beyond those already examined. Undertaking a comprehensive investigation of phase shift effects would be a valuable direction to explore. Furthermore, while this research concentrates on the bidirectional communication topology, it is important to consider other communication topologies such as predecessor–leader following topology, multiple predecessor following topology, etc., as well. Exploring different communication architectures and their implications for platoon dynamics could yield valuable findings. In addition, the present study primarily evaluates the impacts of spacing and velocity difference information on CAV platoons. Nevertheless, incorporating other types of information, such as acceleration, traffic jerks, and electronic throttle opening angle, could provide a more comprehensive analysis of communication side impacts on CAV traffic. Moreover, our current analysis is limited to a one-dimensional perspective. Exploring the effects of lateral dynamics, such as left or right turns, on platoon performance represents a promising avenue for future research. Additionally, this research initially concentrated on introducing a theoretical framework. It is essential to expand the studies to include more comprehensive validation processes in subsequent research. Lastly, while this research mainly evaluates traffic performance by examining string stability and rear-end collision risk, there are other aspects that could be explored. Investigating the damping behavior and energy efficiency of CAV platoons, for example, would offer a more holistic assessment of the impacts of communication on CAV traffic.

## Figures and Tables

**Table 1:** Car-following control models considering bidirectional information.

Type of Information Used	Publications
Spacing	[20,21,22,23,24,25,26]
Velocity difference	[27]
Both spacing and velocity difference	[19,28,29,30,31,32]

**Table 2:** Impact of spacing information on platoon safety and stability.

Control Model	Spacing Information	Rear-End Collision Risk	String Stability
In-Phase Effect	$f_{s_{n+1}}<0$	Decreased	Improved
Opposite-Phase Effect	$f_{s_{n+1}}>0$	Increased	Worsened

**Table 3:** Impact of velocity difference information on platoon safety and stability.

Control Model	Velocity Difference Information	Rear-End Collision Risk	String Stability
In-Phase Effect	$f_{\Delta v_{n+1}}>0$	Decreased	Worsened
Opposite-Phase Effect	$f_{\Delta v_{n+1}}<0$	Increased	Improved

**Table 4:** Parameters used in the IDM model.

$a\;(m\;*\;s^{-2})$	$b\;(m\;*\;s^{-2})$	$v_{d}\;(km\;*\;h^{-1})$	$s_{0}\;(m)$
1	2	120	2

**Table 5:** Parameters in Helly’s model.

Initial Speed (m/s)	$\lambda_{x}$	$\lambda_{v}$	τ
15	1	1	0.8

## Data Availability

The data that support the findings of this study are available on request from the corresponding author.
